# Unveiling the prognostic significance of SOX5 in esophageal squamous cell carcinoma: a comprehensive bioinformatic and experimental analysis

**DOI:** 10.18632/aging.204924

**Published:** 2023-08-02

**Authors:** Chenglin Li, Jialing Zhang, Yanwen Bi

**Affiliations:** 1 Department of Cardiothoracic Surgery, Qilu Hospital of Shandong University, Jinan 250012, Shandong, China; 2 Department of Cardiothoracic Surgery, The Affiliated Huaian No.1 People’s Hospital of Nanjing Medical University, Huaian 223300, Jiangsu, China; 3 Department of Gastroenterology, The Affiliated Huaian No. 1 People’s Hospital of Nanjing Medical University, Huaian 223300, Jiangsu, China

**Keywords:** bioinformatic analysis, SOX5, esophageal squamous cell carcinoma, prognosis

## Abstract

Background: This study aimed to investigate the expression and prognostic significance of SOX5 in esophageal squamous cell carcinoma (ESCC).

Methods: Gene Expression Omnibus (GEO) data were analyzed to assess SOX5 expression in ESCC and normal tissues. Survival analysis was performed to evaluate its prognostic significance. Pathway enrichment analysis was conducted to identify pathways associated with low SOX5 expression. Methylation status of CpG sites in ESCC cases was examined, and SOX5 expression was evaluated. Differential expression and ChIP-seq data analyses were used to identify genes significantly correlated with SOX5 and to obtain target genes. A protein-protein interaction (PPI) network was constructed using hub genes, and their association with immune cell infiltration was determined. *In vitro* ESCC cell experiments validated the findings.

Results: SOX5 was significantly downregulated in ESCC samples compared to normal samples. Its downregulation was associated with shorter survival in ESCC patients. Pathway enrichment analysis revealed enrichment in regulated necrosis, NLRP3 inflammasome, formation of the cornified envelope, and PD-1 signaling. Methylation status of two CpG sites negatively correlated with SOX5 expression. Differential expression analysis identified 122 genes significantly correlated with SOX5, and 28 target genes were obtained from ChIP-seq analysis. Target genes were enriched in DNA replication, cell cycle, spindle, and ATPase activity. Five hub genes were identified, and the PPI network showed significant associations with immune cell infiltration. *In vitro* experiments confirmed SOX5 downregulation, upregulation of hub genes, and their functional effects on ESCC cell apoptosis and proliferation.

Conclusions: These findings enhance understanding of SOX5 in ESCC and potential therapeutic strategies.

## INTRODUCTION

Esophageal cancer (EC) is a common cancer with high mortality rate, and esophageal squamous cell carcinoma (ESCC) is the main histological subtype of EC [1, 2]. As a result of the lack of early disease signs, ESCC is characterized by high prevalence and morbidity [3]. At present, the commonly used treatments are chemotherapy and radiotherapy. However, due to frequent recurrence, limited molecular markers and treatment options, its prognosis is not ideal [4]. Therefore, it is necessary to identify specific molecular markers for the treatment and prognosis of ESCC.

Current research on prognostic markers for esophageal squamous cell carcinoma (ESCC) has made significant progress, aiming to improve patient stratification, prognosis prediction, and treatment outcomes [4]. Multiple studies have focused on identifying molecular markers associated with ESCC prognosis [5]. These markers include genetic alterations, gene expression profiles, protein expression levels, and epigenetic modifications [5]. Various promising prognostic markers have been identified, such as altered expression of specific genes (e.g., TP53, EGFR, and HER2), aberrant DNA methylation patterns, and microRNA signatures [6]. Additionally, the integration of multiple markers or the development of risk prediction models has been explored to enhance prognostic accuracy. The use of high-throughput technologies, such as next-generation sequencing and gene expression profiling, has facilitated the discovery of novel markers with potential clinical utility. However, further validation in large patient cohorts and standardized assessment protocols are necessary to establish the clinical utility of these markers and facilitate their translation into routine clinical practice, ultimately leading to personalized treatment strategies and improved outcomes for ESCC patients.

Transcription factors act an important regulatory part to the life cycle of cells and organisms. They control the transcription rates of genetic information and thus regulate gene expression by specific DNA sequences [7, 8]. SRY-related high-mobility-group box 5 (SOX5) [9] encodes the member of the HMG-box family responsible for maintaining normal physiological functions [10, 11]. Early studies found that SOX5 is dedicated to embryonic development and the cell fate [11]. Recent studies have found that SOX5 dysregulation are associated with cancer [12, 13]. SOX5 is significantly regulated in breast cancer (BC) cell lines, and promoted progress of BC cells [14]. In addition, SOX5 promoted prostate cancer (PC) metastasis, and has been verified *in vivo* [15]. Interestingly, other recent studies have suggested that SOX5 inhibited gliomas progression [16, 17] by acute cellular senescence. Meantime, SOX5 regulates the growth of Kaposi’s sarcoma associated herpesvirus-infected cells [18]. The above findings all imply that SOX5 might have specific functions on cancer.

Our study determined the expression of SOX5 in ESCC using bioinformatic analysis. In addition, we also identified and studied the related hub genes of SOX5 in ESCC. SOX5 and these genes may affect the malignant progression, prognosis and immune infiltration of ESCC.

## MATERIALS AND METHODS

### GEO datasets

GEO (http://www.ncbi.nlm.nih.gov/geo) contains microarrays, chips and gene expression data [19]. GSE23400 (53 ESCC samples and 53 matched normal samples) was used in this study. All downloaded data were matched with clinical information, standardized and log2 translated.

### TCGA datasets

The RNA-seq data of SOX5 and the clinical information of TCGA-ESCC were from the UCSC Xena browser (https://xenabrowser.net). The samples data including the information of age, gender, clinical stage, lymph node metastasis, neoplasm histologic grade and overall survival (OS) were analyzed. Furthermore, the samples were divided into two groups in line with median SOX5 expression, Gene Set Enrichment Analysis (GSEA) was conducted. At the same time, the sample contains RNA-seq data of SOX5 and methylation data were filtered. The Champ package was used to pre-process the methylation data, and the CpG sites in the SOX5 promoter region (TSS200, TSS1500) were selected. The correlation between the beta value of these CpG sites and SOX5 expression was calculated using Pearson analysis. CpG sites with SNP were ignored.

### Identification of DEGs

DEGs between ESCC and normal samples were identified by Limma package (|logFC|>1 and *p*-value<0.05). First, we preprocessed the data to remove biases and normalize the expression values. Next, we constructed a design matrix to model the experimental design and define the comparisons of interest. Using this design matrix, a linear model was fitted to the data, considering the experimental factors and estimating treatment effects. We then defined contrasts to specify the desired comparisons between conditions. Statistical testing was performed using moderated t-statistics to evaluate the significance of differential expression. Multiple testing correction was applied, and DEGs were identified based on adjusted p-values and fold change thresholds. The resulting DEG list was annotated with relevant information, such as gene symbols and pathways. Finally, visualizations and analyses were generated to aid in the interpretation of the DEGs. Based on Pearson correlation analysis, the correlation of DEGs and SOX5 expression was analyzed.

### ChIP-seq data analysis

The results of bed peaks of ChIP-seq data for SOX5 were provided by Cistrome DB (http://cistrome.org/db/#/). The downloaded narrow peak data were annotated and filtered with a threshold value of Q-value and IDR < 0.01. The peaks within the range of 1 kb upstream or downstream of transcription start site (TSS) were retained, and the genes on these peaks were defined as target genes. Dreme was used to identify significant motif sequences in peak sequences.

### Enrichment analysis

Biological functions of target genes were conducted by clusterProfiler package in Bioconductor. Gene Ontology (GO), Kyoto Encyclopedia of Genes and Genomes (KEGG), and Gene Set Enrichment Analysis (GSEA) were performed to identify enriched biological processes, molecular functions, cellular components, and pathways associated with a set of genes of interest. GO enrichment analysis compared the genes against a background set using statistical tests and adjusted p-values or false discovery rate (FDR) correction. Similarly, KEGG enrichment analysis compared genes to KEGG pathways, while GSEA ranked genes and evaluated enrichment scores through permutation testing. Enriched terms and pathways were identified based on predefined thresholds, providing insights into gene function and pathway associations.

### Protein-protein interaction (PPI) network construction

STRING (http://string-db.org, version 11.0) database was used to construct the PPI network of target genes. Next, we use Cytoscape software to visualize the protein-protein interaction network and describe the relationship between different proteins by line segments, in which the more line segments represented the more significant the interaction between proteins.

### Hub genes selection and analysis

Nodes were ranked in the PPI network with respect to their network features through Cytoscape, which is the cytoHubba plug-in [20]. Here, the maximal clique centrality (MCC), and edge percolated component (EPC) were applied as the criteria for ranking the top 5 hub genes. The overlapping top 5 genes in these two topological methods were regarded as hub genes for further analysis [21].

### Immune infiltration analysis

The 22 immune cell types in samples were assessed using the CIBERSORT algorithm with the criteria of *p*-value <0.05. The proportion of each kind of immune cell in the samples was calculated. The Wilcoxon rank-sum test was utilized to determine the percentage of immune-infiltrating cells between ESCC and control samples. After inclusion of all significant cells into the Cox model, co-expressed heatmap was drawn by combining with Pearson correlation analysis, and the correlation of various immune cells and hub genes was displayed.

### Cell lines and cell culture

Human ESCC cell lines KYSE150, KYSE30, and normal esophageal epithelial cell lines (Het1A) were cultured in RPMI-1640 with 10% FBS (GIBCO, Waltham, MA, USA) at 37° C under 5% CO_2_.

### Plasmid construction and cell transfection

The full-length of SOX5 cDNA was generated and inserted into the pAd-CMV-MC5 to construct SOX5 overexpression vector (Ad-SOX5) [22]. Lipofectamine 2000 (Thermo Fisher Scientific, Cleveland, OH, USA) was applied for cell transfection.

### Western blot

After lysing, protein of cells was separated by SDS-PAGE and transferred onto PVDF membrane (Millipore, USA). Primary antibodies were incubated on the membrane at 4° C overnight: SOX5 (1:1000, Abcam), PCNA (1:1000, Abcam), RRM2 (1:1000, Abcam), AURKB (1:1000, Abcam), MCM4 (1:2000, Abcam), MCM7 (1:1000, Abcam), GAPDH (1:1000, Abcam). The results were determined with a chemiluminescence kit (Thermo Fisher Scientific, Cleveland, OH, USA).

### Cell proliferation assay

For CCK-8 assay, treated cells in 96-well culture plates (1.5 × 10^3^ cells/well) were treated with 10 μL CCK-8 solution (Dojindo, Kumamoto, Japan) at different time points. After 2 h, the optical density (OD) at 450 nm was detected.

For EdU assay, treated cells in 96-well plates (1.5 × 10^4^ cells/well) were incubated with EdU working solution (Cat.C10310-1, Ruibo Biotech., Guangzhou, China) for 2 h. After fixation, permeabilization, cells were stained with 1x Apollo solution. Results were obtained under fluorescence microscope.

### Flow cytometry

Annexin V-FITC apoptosis detection kit (Oncogene Research Products, Boston, MA, USA) was used to determine the apoptosis of cells.

### Statistical analysis

R V4.0.2 was used to perform statistical analysis. Survival rates were determined by Kaplan-Meier survival curves. Pearson’s correlation test was utilized to detect the extent of correlation between the expression of SOX5 and the methylation/DEGs, and hub genes. The differences between two groups were analyzed by GraphPad Prism 8. *p*< 0.05 indicates a statistically significant difference.

## RESULTS

### Low expression of SOX5 in ESCC is associated to survival

**Figure 1:**
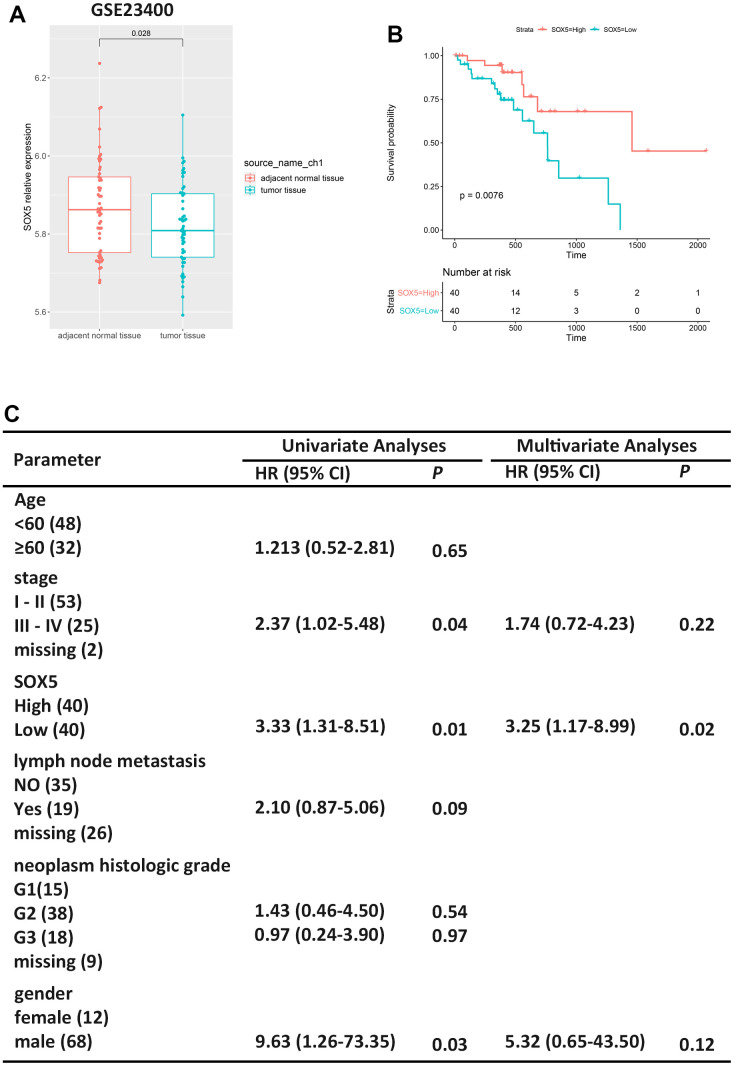
**Low expression of SOX5 in ESCC is associated to survival.** (**A**) Expression boxplots of SOX5 in ESCC tumor tissue and adjacent normal tissue from GSE23400. (**B**) Survival analysis of SOX5 in ESCC samples from TCGA-ESCC. (**C**) Univariate and multivariate cox regression analyses of clinicopathological characteristics for OS in ESCC samples from TCGA-ESCC. HR, hazard ratio; CI, confidence interval.

To explore the SOX5 expression in human ESCC tissues, we downloaded microarray gene profiling datasets GSE23400 (53 ESCC samples and 53 matched normal samples) from the GEO. We analyzed SOX5 expression in datasets, and the results are shown in Figure 1A, indicating that SOX5 was downregulated in ESCC tumor tissue compared with normal tissue (*p*=0.028). The results indicated that SOX5 might play an important role in ESCC.

Therefore, we screened 80 ESCC samples in TCGA-ESCC from the XENA. First, the relationship between SOX5 expression and survival outcomes in the ESCC cohort was evaluated (Figure 1B, *p*=0.0076). Furthermore, SOX5 low expression group had a shorter survival time than the high expression group. Additionally, univariate analysis and multivariate analyses of Cox regression model were performed. In univariate analysis, low SOX5 expression and gender were significantly correlated to OS of ESCC patients (Figure 1B). In multivariate analysis, low SOX5 expression was significantly correlated to OS in patients with ESCC (Figure 1C). The results of COX regression are shown in Supplementary Figure 2.

### Results of GSEA in ESCC

**Figure 2:**
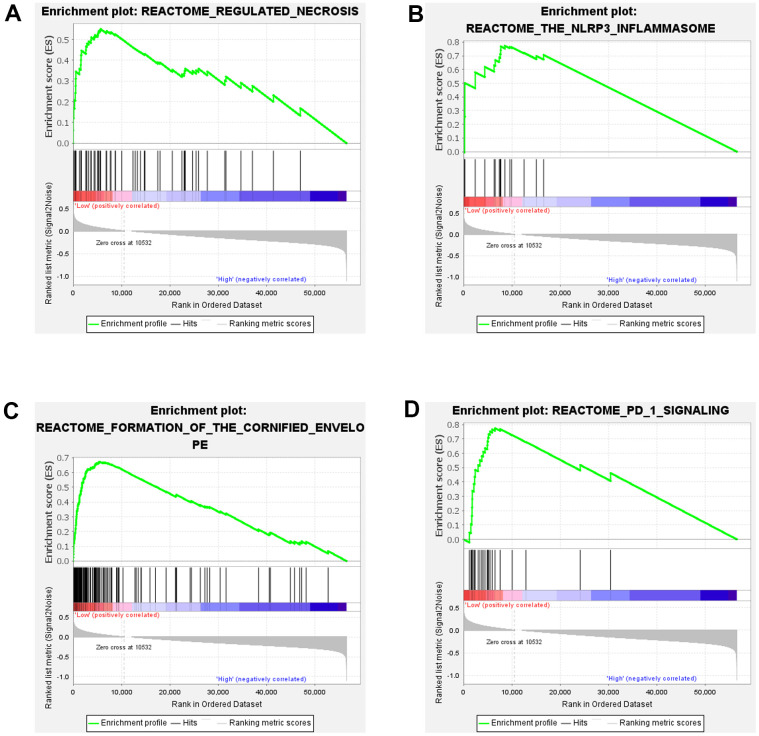
**Results of GSEA in ESCC.** (**A**–**D**) GSEA enrichment analysis of SOX5 in TCGA-ESCC.

To gain a deeper understanding of the biological functions of SOX5 in ESCC, we conducted GSEA. Similarly, the samples from TCGA-ESCC cohort were divided into two groups in line with the median SOX5 expressions in the cohort as a threshold. As the results showed, low expression of SOX5 group was mainly enriched in the pathway of regulated necrosis, the NLRP3 inflammasome, formation of the cornified envelope and PD 1 signaling (Figure 2A–2D).

### SOX5 expression is negatively correlated with its methylation in ESCC

**Figure 3:**
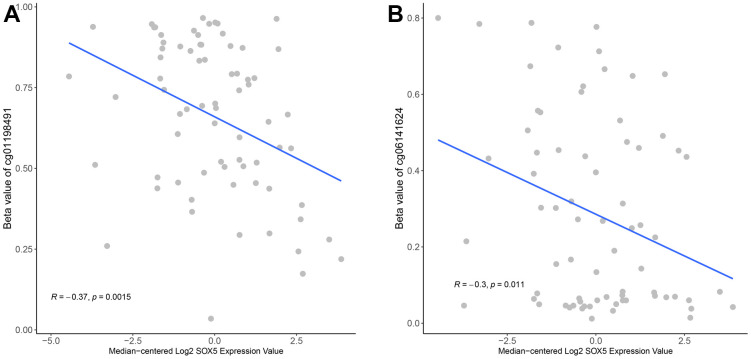
**SOX5 expression was negatively correlated with its methylation in ESCC.** The correlation of SOX5 expression and beta-value of cg01198491 (**A**) and cg06141624 (**B**).

To understand the potential regulatory effect of methylation on SOX5 expression, we downloaded the TCGA-ESCC methylation chip data from XENA. After filtering, 74 ESCC samples with both RNA-Seq data of SOX5 and methylation data were obtained. The CpG sites on the SOX5 gene were filtered, the CpG sites located in the promoter region were retained, and 57 CpG sites were obtained. The association between SOX5 expression and the β-value of CpG sites was identified using the Pearson’s correlation analysis. Results (Figure 3A, 3B) indicated a negative correlation between SOX5 expression and 2 sites: cg01198491 (Pearson R=-0.37; *p*<0.01), and cg06141624 (Pearson R= -0.3; p<0.05).

### Identification of DEGs in ESCC

**Figure 4:**
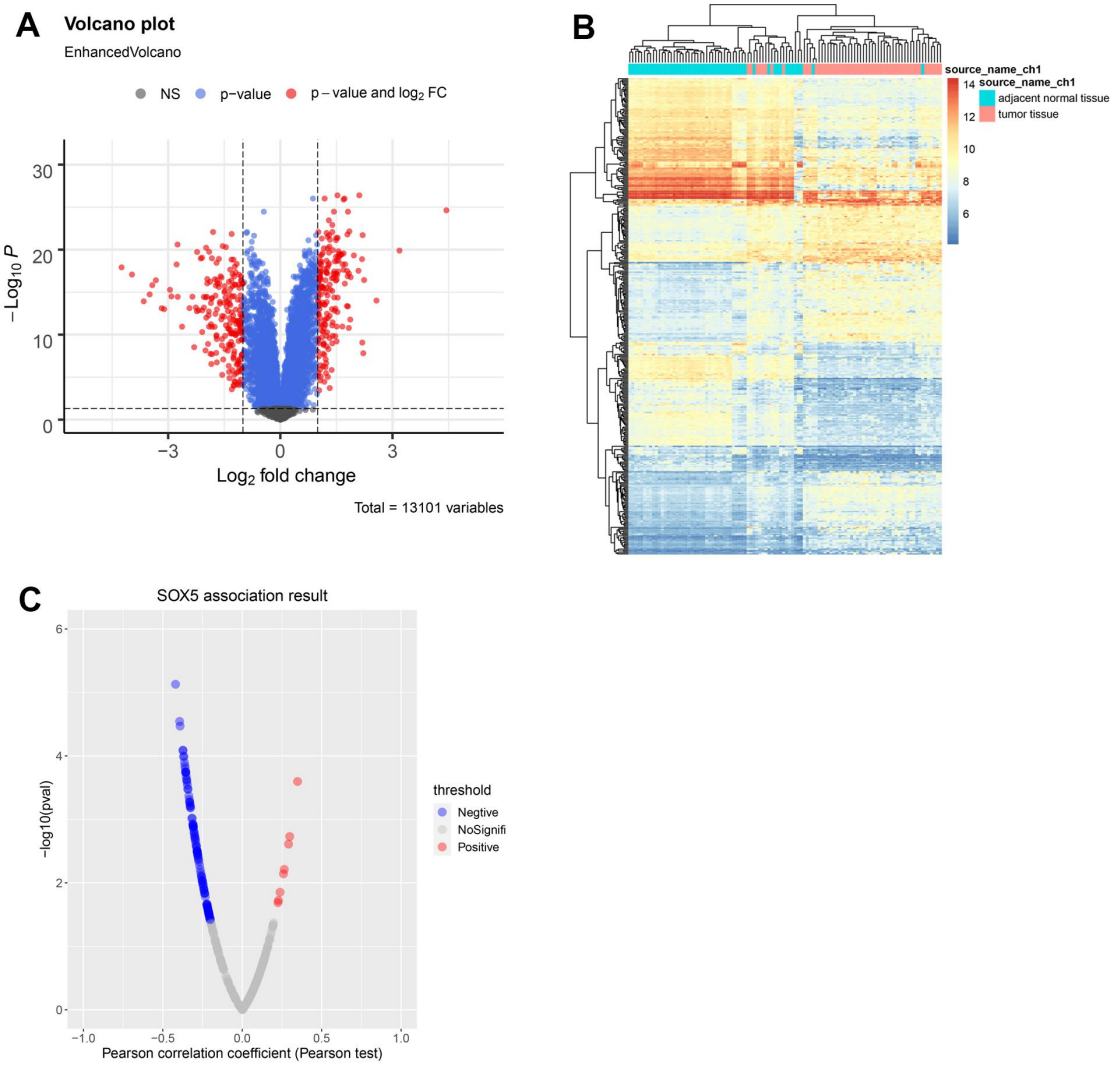
**Identification of DEGs in ESCC.** (**A**) Volcano map and (**B**) heatmap of DEGs between ESCC samples and control samples from GSE23400. (**C**) Correlations between SOX5 and DEGs in ESCC were analyzed using Pearson-correlation analysis.

Via Limma, DEGs in the GSE23400 dataset were identified (Figure 4A, 4B). By screening, 187 upregulated and 207 downregulated DEGs were obtained. The results were presented with the volcano map and heatmap. The correlation between the above DEGs and SOX5 was calculated by Pearson correlation coefficient with a PCC > 0.2 and a *p*-value < 0.05. Figure 4C shows that 122 genes were significantly correlated with SOX5.

### ChIP-seq data analysis of target genes of SOX5

**Figure 5:**
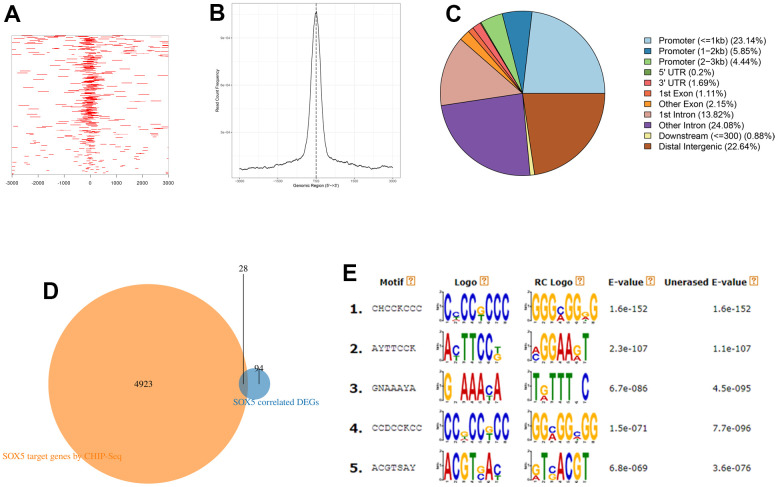
**ChIP-seq analysis of target genes of SOX5.** (**A**) Heatmap of ChIP binding to TSS regions. (**B**) Average tag density profiles. (**C**) Pie plot of genomic regions of peaks. (**D**) The intersection of the results of ChIP-seq data analysis and significantly correlated DEGs. (**E**) Top5 predicted motifs by DREME.

To further explore the correlated genes of SOX5, we predicted target genes of SOX5 using ChIP-seq data assay and predicted the SOX5 binding sites. Distribution of binding peaks in the SOX5 ChIP-seq experiment from Cistrome DB were analyzed. The peaks in the range of I kb of upstream and downstream of TSS were reserved. Approximately 6324 peaks were located on 4951 genes (Figure 5A–5C). We took the intersection of these genes and 122 significantly correlated DEGs, and finally 28 target genes were obtained (Figure 5D). And DREME (https://meme-suite.org/meme/tools/dreme) online software was used to predicted SOX5 binding motifs (Figure 5E).

### Gene ontology and pathway functional enrichment analysis

**Figure 6:**
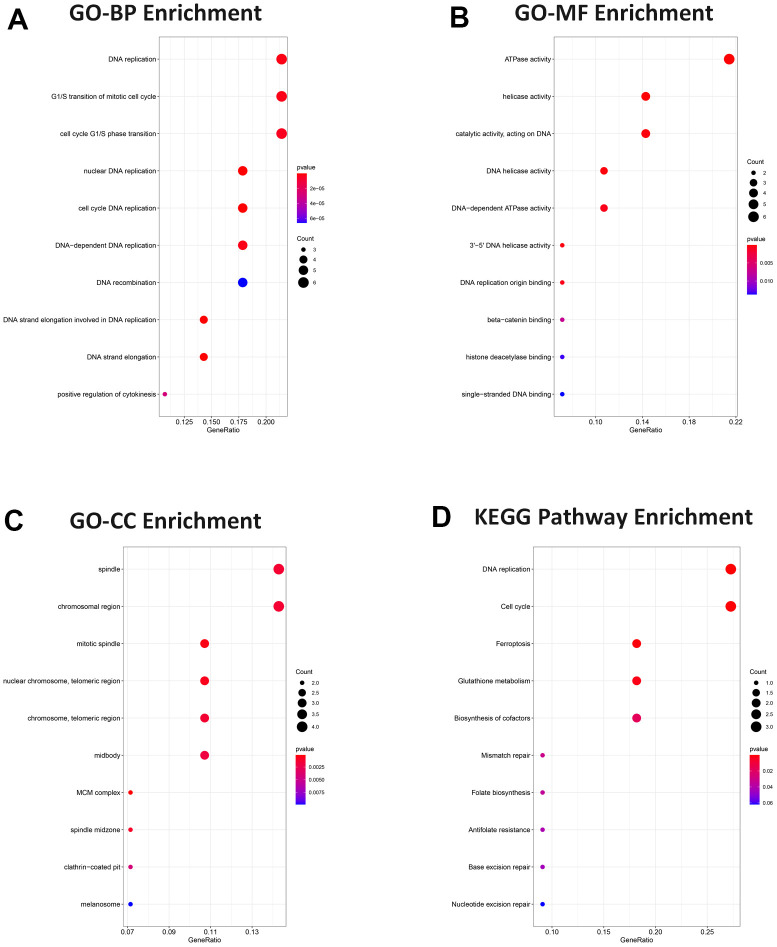
**Gene ontology and pathway functional enrichment analysis.** (**A**) BP analysis. (**B**) MF analysis. (**C**) CC analysis. (**D**) KEGG pathway. Functional and pathway enrichment were presented in bubble charts, ranking the top 10 according to adjusted P value.

A total of 28 target genes were conducted with GO and KEGG enrichment analysis. Genes were enriched in DNA replication, G1/S transition of mitotic cell cycle, and cell cycle G1/S phase transition in the biological process (BP) analysis (Figure 6A). For molecular function (MF) analysis, genes were primarily linked to the ATPase activity, helicase activity, and catalytic activity, acting on DNA (Figure 6B). The enrichment analysis of cell component (CC) indicated that genes were related to spindle, chromosomal region, and mitotic spindle (Figure 6C). For KEGG enrichment analysis, genes were enriched mainly in DNA replication, cell cycle and ferroptosis (Figure 6D). These analyses suggested that target genes had a crucial effect on ESCC.

### Hub genes identification

**Figure 7:**
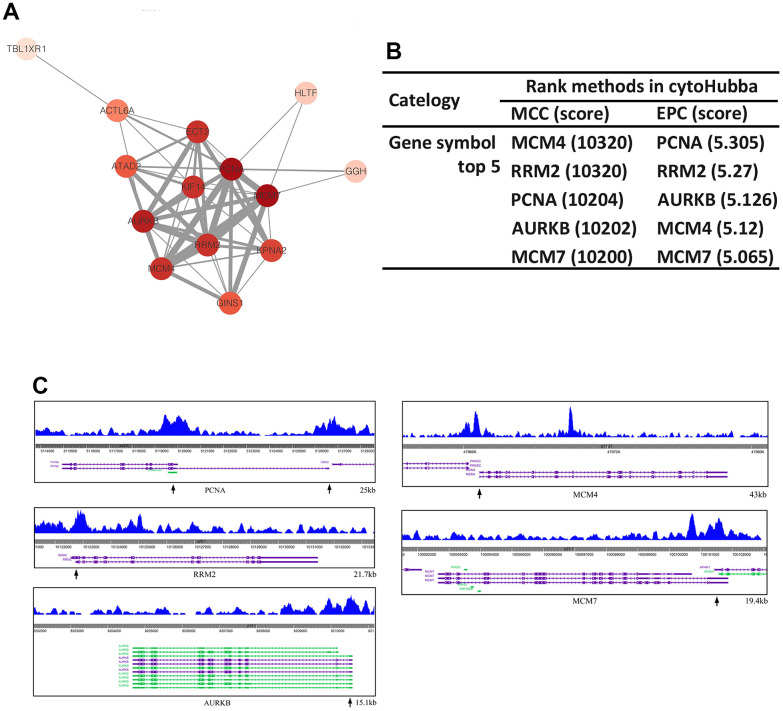
**Hub genes identification.** (**A**) The PPI network of DEGs was established by STRING. (**B**) The top 5 nodes of PPI network evaluated in cytoHubba MCC and EPC arithmetic. (**C**) ChIP-seq profiles of SOX5 at promoter region of PCNA, RRM2, AURKB, MCM4, MCM7.

The potential relationships among 28 target genes were detected by STRING database, and PPI network was established (minimum required interaction score > 0.4) (Figure 7A). Next, two topological analysis methods, MCC and EPC, were used to rank the top 5 genes in the PPI network (Figure 7B). The results showed that 5 overlapping hub genes (PCNA, RRM2, AURKB, MCM4 and MCM7) were identified. And ChIP-seq profiles of SOX5 at promoter region of hub genes were presented in Figure 7C.

### Pathway enrichment analysis of hub genes

**Figure 8:**
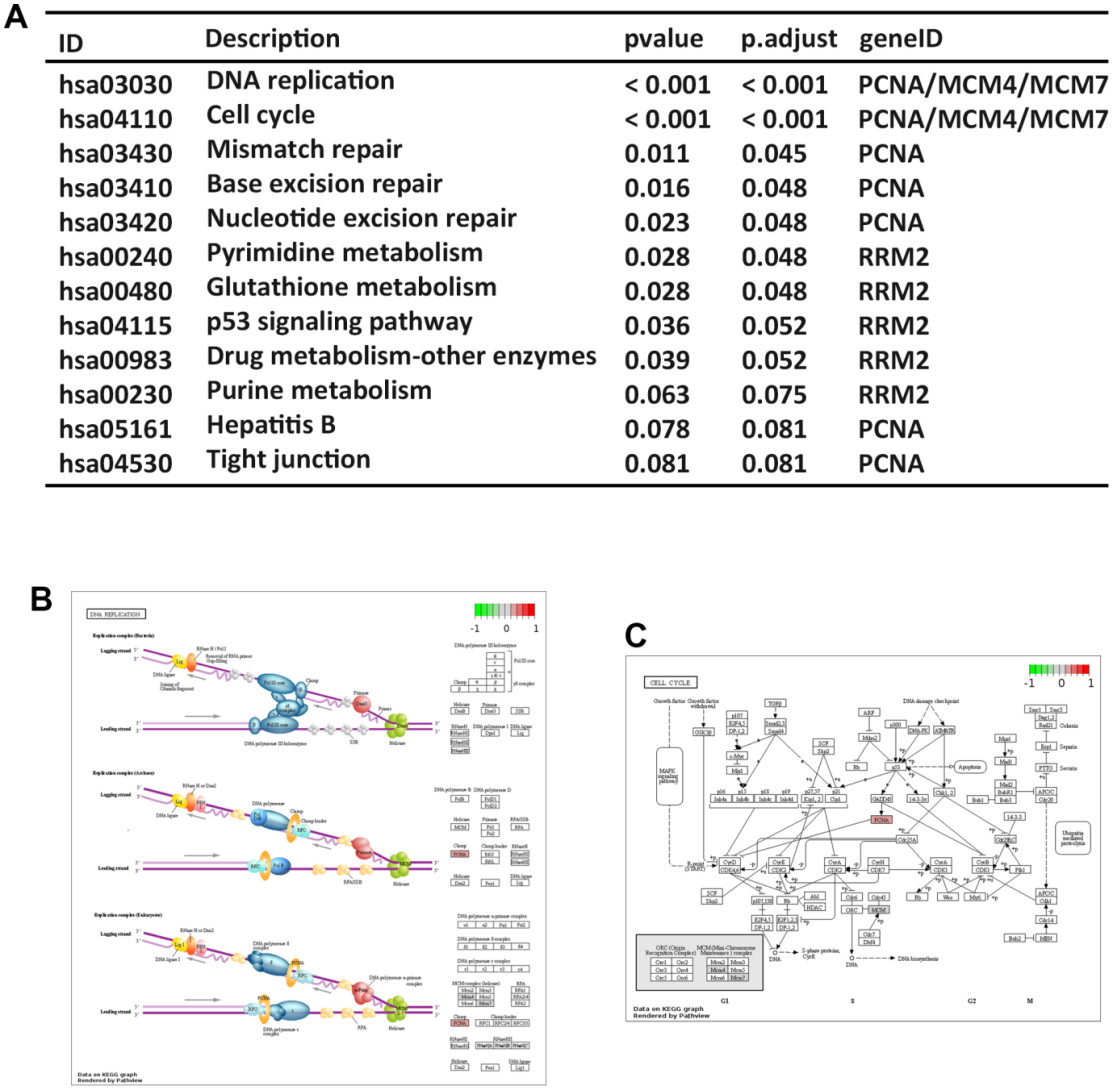
**Pathway enrichment analysis of hub genes.** (**A**) KEGG pathway analysis. KEGG pathway annotations of DNA replication signaling (**B**) and cell cycle signaling pathways (**C**).

The expressions of 5 hub genes were significantly higher in ESCC samples compared to normal samples (Supplementary Figure 1). The results of pathway analysis (Figure 8A) showed that 3 hub genes (PCNA, MCM4 and MCM7) were related to DNA replication and cell cycle pathway (Figure 8B, 8C). These findings are consistent with the molecular pathways implicated in ESCCC carcinogenesis. Further, the previous study indicated that the elevated PCNA+ tumor-associated macrophages in early recurrence of BC [23]. MCM7 has been found to be involved in the immune response of the spleen, thereby influencing tumor development [24]. These results implied that hub genes involve in the tumor progression and immune response.

### Immune infiltration analysis

**Figure 9:**
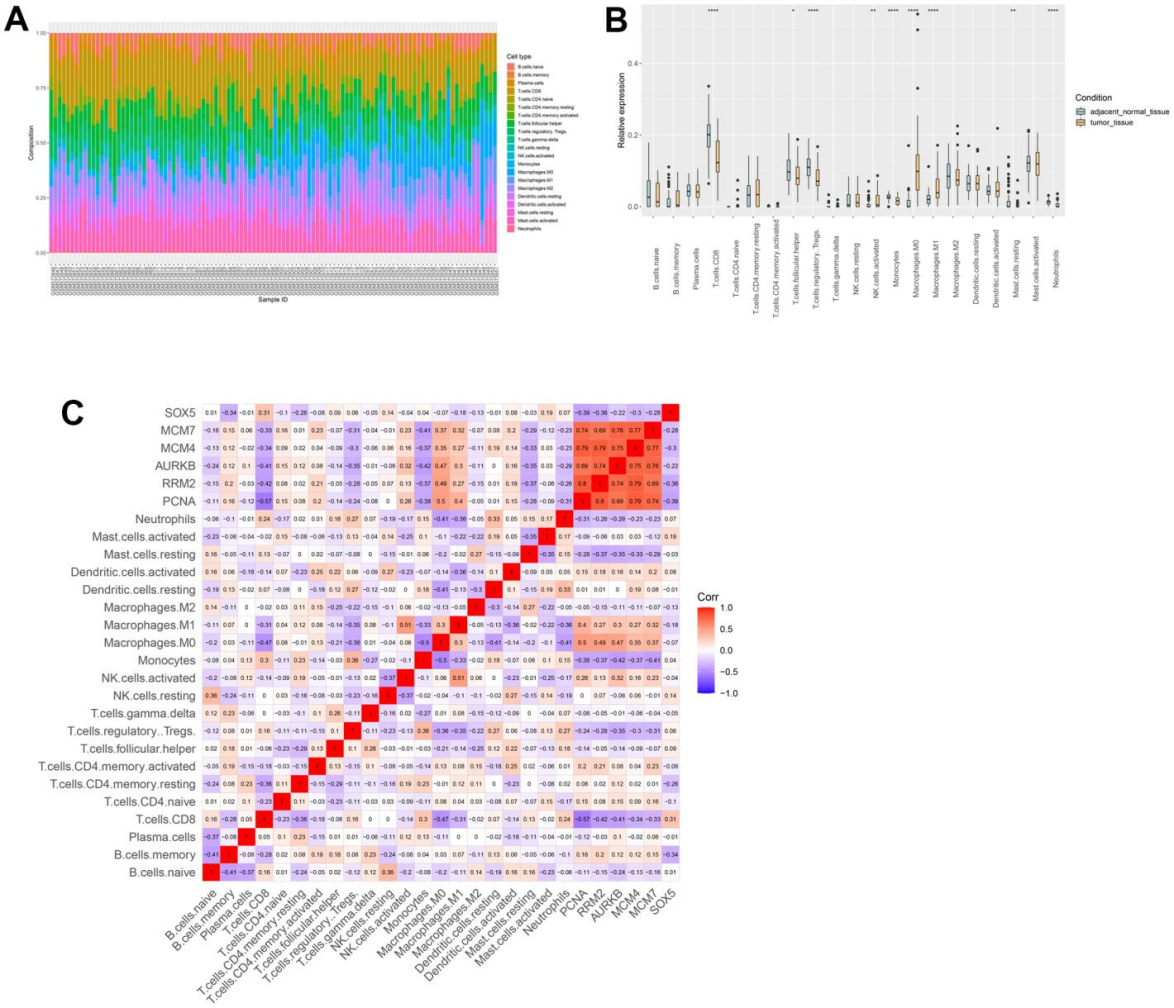
**Immune infiltration analysis.** (**A**) The composition of immune cells estimated in ESCC. (**B**) Differential expression of different types of immune cells between ESCC and normal samples. (**C**) The relationships among immune cells and between hub genes and immune cells.

Therefore, we investigated whether hub genes were correlated with immune infiltration levels in ESCC. The composition of 22 immune cells (Figure 9A) and the distribution of immune cells (Figure 9B) in samples uncovered an important relationship between ESCC and immune cell infiltration. In addition, Figure 9C shows that there is a noteworthy positive correlation between 5 hub genes and the infiltration of Macrophages.M0 and Macrophages.M1.

### Upregulation of SOX5 inhibited proliferation and promoted apoptosis of ESCC cells *in vitro*

**Figure 10:**
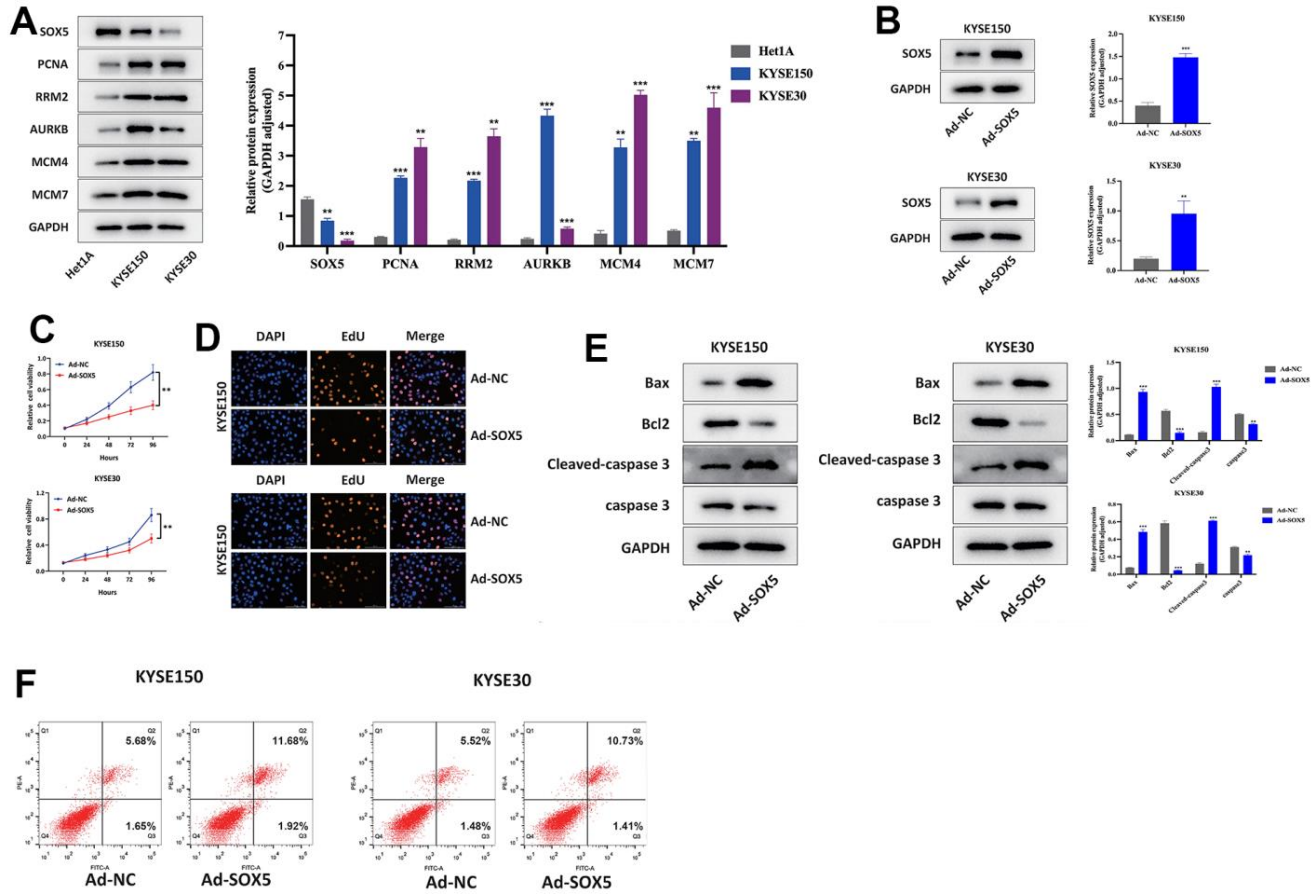
**Upregulation of SOX5 inhibited proliferation and increased apoptosis of ESCC cells *in vitro*.** (**A**) Western blot assay of SOX5 and hub genes expression in ESCC and Het1A cells. (**B**) Western blot assay of SOX5 protein in control and overexpression ESCC cells. (**C**, **D**) CCK-8 assay (**C**) and EdU assay (**D**) results of different treated cells. (**E**) Western blot assay of Bcl2, Bax, cleaved-caspase 3 and caspase 3 protein in different treated cells. (**F**) Flow cytometry analysis of apoptosis of different treated cells. *P < 0.05, **P < 0.01.

We sought to elucidate the expression of SOX5 and 5 hub genes through studies on ESCC cells *in vitro*. Using Western blot, SOX5 was downregulated and 5 hub genes were upregulated in different ESCC cell lines, compared to Het1A cells (Figure 10A). Furthermore, SOX5 in ESCC cell lines was overexpressed (Figure 10B). Subsequently, upregulation of SOX5 showed inhibitory effects on ESCC cell proliferation (Figure 10C, 10D). Flow cytometric analysis and Western blot showed Upregulation of SOX5 elicited apoptosis of KYSE30 and KYSE150 cells (Figure 10E, 10F) (*p<0.05, **p<0.01, ***p<0.001).

## DISCUSSION

This study identified that SOX5 was significantly downregulated in ESCC samples than in the control. And SOX5 downregulation was significantly related to a poor prognosis. In addition, the survival time was significantly shorter in the SOX5 low expression group compared to high expression group. And combined with the results of GSEA analysis, we speculated that SOX5 might serve as a significative prognostic biomarker for ESCC.

Previous studies have indicated that SOX5 expression depends on multiple genomic and epigenetic mechanisms in embryonic development, such as methylation [11, 25, 26]. In the present study, SOX5 methylation status was also determined in 74 ESCC samples. SOX5 expression was negatively correlated with 2 CpG sites. However, due to complexity of epigenetic mechanisms, we also need to conduct in-depth research in the future, such as heterozygous point mutations and genomic deletions [27].

So far, the function of SOX5 in different cancers is quite different. In general, the function of transcriptional factors depends mainly on their downstream target genes [15, 22, 28, 29]. Therefore, we explored the potential upstream or downstream genes of SOX5 in ESCC. By screening the DEGs, and then significantly correlated DEGs were obtained by correlation analysis with SOX5 expression. Followed by ChIP-seq data analysis, potential target genes were identified by intersecting with the above genes. PPI network was constructed, and combined with MCC and EPC algorithms, 5 hub genes were identified. Among them, genes have well-characterized tumor-promoting effects.

Proliferating cell nuclear antigen (PCNA) acts an important part to DNA replication and repair [30]. PCNA promotes tumor growth of gastric cancer (GC) [31]. Inhibition of tyrosine phosphorylation of PCNA suppress PC growth [32]. Ribonucleotide reductase small subunit M2 (RRM2) is important in promoting EMT in PC cells, leading to a poor prognosis in PC patients [33]. LncRNA AFAP1-AS1 promote non-small cell lung cancer cell proliferation. The specific mechanism is the suppression of miR-139-5p by competitive upregulation of RRM2 through ceRNA network [34]. Aurora kinase B (AURKB) plays a crucial part during mitosis in mammals, and could promote GC [35]. As the member of Minichromosome maintenance protein family, MCM4 is high expressed in esophageal carcinomas [36] and high histological grades BC [37]. And MCM7 was found to promote hepatocellular carcinoma through cyclin D1-dependent signaling [38]. Meantime, high expression of MCM7 is closely related to PC [39].

Moreover, immune infiltration analysis showed that hub genes were positively correlated with the immune cells (macrophages.M0 and macrophages.M1) infiltration. Immune cell infiltration is closely related to tumor progression [40–42] However, the specific mechanism of hub genes related to immune infiltration in ESCC needs to be verified by subsequent experiments in the future.

In conclusion, our study has identified down-regulated expression of SOX5 as a prognostic marker associated with poor prognosis in esophageal squamous cell carcinoma (ESCC). This finding has significant implications for patient management and treatment decisions. The incorporation of SOX5 expression analysis into existing prognostic models may enhance their accuracy, enabling more precise prognostic assessments and risk stratification. Furthermore, the correlation between down-regulated SOX5 expression and poor prognosis suggests its potential as a predictive marker for treatment response. Future research on the functional role of SOX5 in ESCC could lead to the development of targeted therapies and personalized treatment approaches aimed at restoring or enhancing SOX5 expression. The monitoring of SOX5 expression during treatment may also provide insights into treatment response and guide clinical decisions. Overall, our findings highlight the clinical relevance of SOX5 as a prognostic marker in ESCC and pave the way for further investigations in this field. However, several limitations should be considered in interpreting the findings of our study. Firstly, the sample size used in our study was relatively small, which may limit the generalizability of the results. Further validation studies using larger cohorts are warranted to confirm the association between SOX5 down-regulation and prognosis in ESCC. Secondly, the study focused solely on the expression levels of SOX5 and its correlation with prognosis. Additional functional studies, such as *in vitro* and *in vivo* experiments, are needed to elucidate the underlying mechanisms by which SOX5 influences ESCC progression and to assess its potential as a therapeutic target. Moreover, although efforts were made to account for potential confounding factors, unmeasured variables or other molecular alterations in ESCC could influence the observed associations. Finally, the study utilized retrospective data, which may introduce inherent biases and limitations in data collection and analysis. Prospective studies with well-defined protocols and standardized assessments would provide more robust evidence regarding the clinical utility of SOX5 as a prognostic marker in ESCC.

## CONCLUSIONS

In our study, we finally verified SOX5 and its target hub genes (PCNA, RRM2, AURKB, MCM4, and MCM7) as significative key factors in ESCC. Furthermore, our study also found a strong relation between the expression of key factors and prognosis and immune cell infiltration. These findings may provide further understanding of the tumorigenesis of ESCC based on several potential immune-related targets. In the future, clinical study and further biological experiments are necessary to validate our results.

## Supplementary Material

Supplementary Figures
