# Supplementary material for: Unveiling the prognostic significance of SOX5 in esophageal squamous cell carcinoma: a comprehensive bioinformatic and experimental analysis
Source: Aging (Albany NY). 2023 Aug 2;15(15):7565–82. doi: 10.18632/aging.204924 (PMC10457070; doi:10.18632/aging.204924)
Supplement: Supplementary Figures [file aging-15-204924-s001.pdf]

SUPPLEMENTARY FIGURES

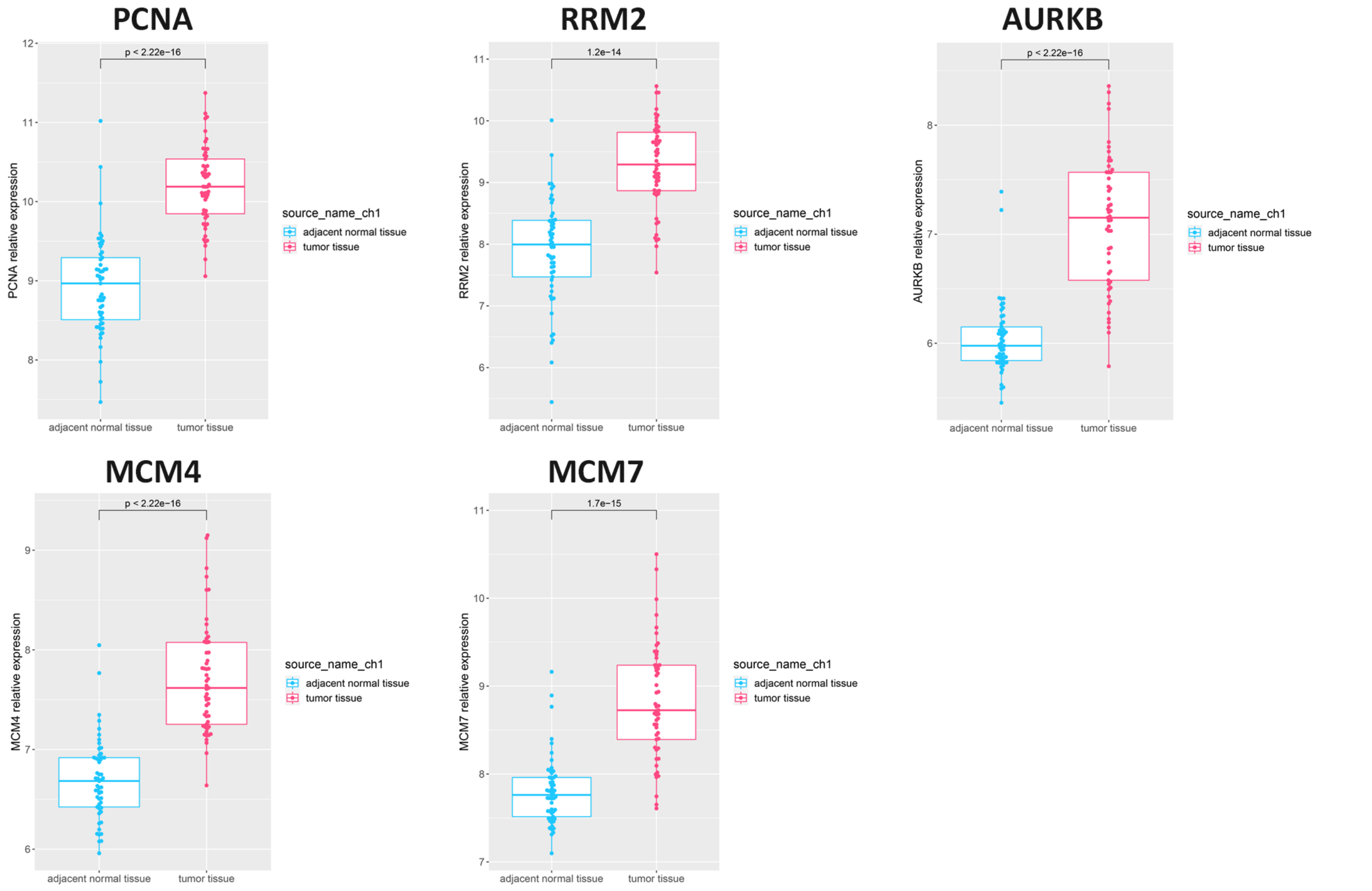

Supplementary Figure 1. Expression boxplots of hub genes in ESCC samples and matched normal samples from GSE23400.

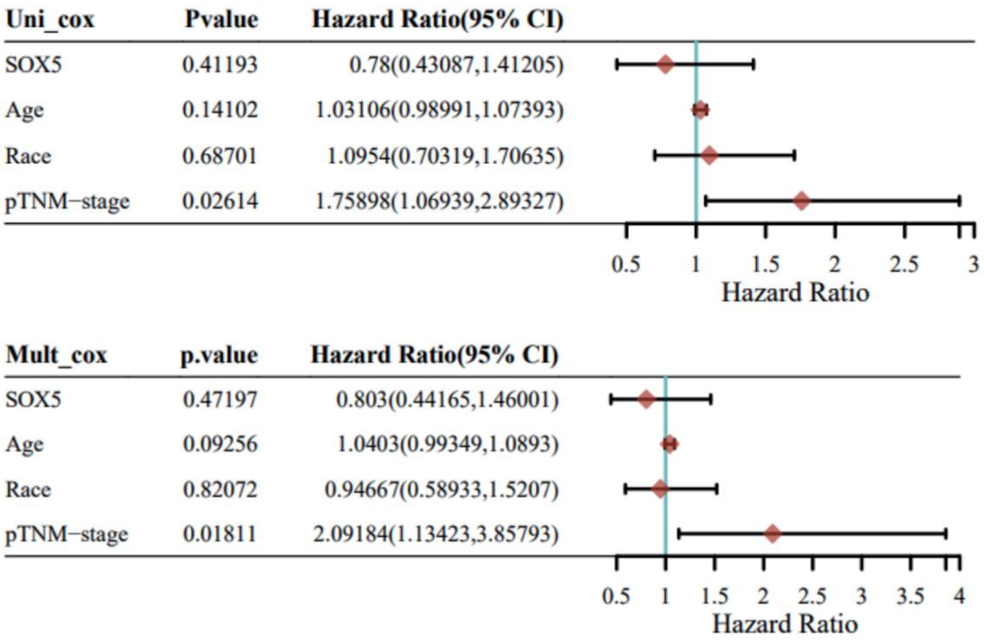

Supplementary Figure 2. The Cox regression.
